# Detection of T Wave Peak for Serial Comparisons of JTp Interval

**DOI:** 10.3389/fphys.2019.00934

**Published:** 2019-07-25

**Authors:** Katerina Hnatkova, Jose Vicente, Lars Johannesen, Christine Garnett, David G. Strauss, Norman Stockbridge, Marek Malik

**Affiliations:** ^1^National Heart and Lung Institute, Imperial College London, London, United Kingdom; ^2^Division of Cardiovascular and Renal Products, Office of New Drugs, Center for Drug Evaluation and Research, U.S. Food & Drug Administration, Silver Spring, MD, United States; ^3^Division of Applied Regulatory Science, Office of Clinical Pharmacology, Office of Translational Sciences, Center for Drug Evaluation and Research, U.S. Food and Drug Administration, Silver Spring, MD, United States

**Keywords:** JTp interval prolongation, T wave peak measurement, one-dimensional ECG representation, intra-subject stability of rate corrected ECG intervals, normal healthy subjects

## Abstract

Electrocardiogram (ECG) studies of drug-induced prolongation of the interval between the J point and the peak of the T wave (JTp interval) distinguished QT prolonging drugs that predominantly block the delayed potassium rectifier current from those affecting multiple cardiac repolarisation ion channel currents. Since the peak of the T wave depends on ECG lead, a “global” T peak requires to combine ECG leads into one-dimensional signal in which the T wave peak can be measured. This study aimed at finding the optimum one-dimensional representation of 12-lead ECGs for the most stable JTp measurements. Seven different one-dimensional representations were investigated including the vector magnitude of the orthogonal XYZ transformation, root mean square of all 12 ECG leads, and the vector magnitude of the 3 dominant orthogonal leads derived by singular value decomposition. All representations were applied to the median waveforms of 660,657 separate 10-s 12-lead ECGs taken from repeated day-time Holter recordings in 523 healthy subjects aged 33.5 ± 8.4 years (254 women). The JTp measurements were compared with the QT intervals and with the intervals between the J point and the median point of the area under the T wave one-dimensional representation (JT50 intervals) by means of calculating the residuals of the subject-specific curvilinear regression models relating the measured interval to the hysteresis-corrected RR interval of the underlying heart rate. The residuals of the regression models (equal to the intra-subject standard deviations of individually heart rate corrected intervals) expressed intra-subject stability of interval measurements. For both the JTp intervals and the JT50 intervals, the curvilinear regression residuals of measurements derived from the orthogonal XYZ representation were marginally but statistically significantly lower compared to the other representations. Using the XYZ representation, the residuals of the QT/RR, JTp/RR and JT50/RR regressions were 5.6 ± 1.1 ms, 7.2 ± 2.2 ms, and 4.9 ± 1.2 ms, respectively (all statistically significantly different; *p* < 0.0001). The study concludes that the orthogonal XYZ ECG representation might be proposed for future investigations of JTp and JT50 intervals. If the ability of classifying QT prolonging drugs is further confirmed for the JT50 interval, it might be appropriate to replace the JTp interval since with JT50 it appears more stable.

## Introduction

This article concerns different methodologies for the detection of the peak of the T wave in a 12-lead electrocardiogram (ECG) with the aim of serving serial comparisons of the J to T peak (JTp) interval. By this we mean situations when it needs to be investigated whether the JTp interval was changed during a certain intervention. As explained further, such situations emerge during pharmacologic investigations of repolarisation-active drugs. Nevertheless, other needs for serial JTp comparisons might also emerge, e.g., in physiologic studies of exposure to environmental changes, physical exhaustion, autonomically active provocations, etc. All such situations lead to the comparison of ECGs obtained before and after the intervention. Since the investigated intervention might also change heart rate, stability of the JTp relationship to underlying heart rate is needed which, in turn, might depend of the method for the T peak detection.

Superficially, it might seem that T wave peak is easier to measure compared to the end of the T wave. Nevertheless, this belief is not justified, especially if considering the need for serial comparisons of JTp intervals in ECGs recorded under different conditions. While in physiologic ECGs that do not show any repolarisation abnormality, the end of the T wave is the same in different ECG leads (apart from those leads in which the terminal part of the T wave is projected onto the isoelectric line) (Malik, 2004), T wave peak is determined by lead-specific projection of the vectorcardiographic T wave loop and frequently differs lead to lead (Malik et al., 2018). Moreover, in serial comparisons, constant orientation of the heart in the thorax and thus the same projection of the T wave loop cannot be expected. Therefore, morphological information of different ECG leads need to be combined into a signal matrix that reasonably represents the T wave loop and that allows reproducible and stable detection of the T peak.

### Importance in Pharmacological Investigations

The drug-induced QTc interval prolongation is caused by all pharmaceutical compounds that lead to proarrhythmia due to the induction of Torsade de Pointes tachycardia (Fenichel et al., 2004). Therefore, the testing of drug-induced QTc changes belongs to regulatory evaluation of all new pharmaceuticals (Guidance to industry, 2005). However, while the test of torsadogenic toxicity based on QTc prolongation has high sensitivity (i.e., drugs causing Torsade de Pointes also prolong the QTc interval) its specificity is not so high (i.e., not all QTc prolonging drugs are proarrhythmic) (Fenichel et al., 2004).

For this reason, different paradigms of proarrhythmic drug testing are presently being discussed (Vicente et al., 2016, 2018; Strauss et al., 2018). These include classification of drugs along their actions on myocardial ion channels. Among others, differentiation is needed between drugs that cause QTc interval prolongation by predominantly blocking the delayed potassium rectifier current (IKr) and drugs for which the IKr blockade is mitigated by effects on inward currents active during myocardial repolarisation.

In ECG studies of drugs with known effects on myocardial ion channels, it has been observed that drugs that prolong the QT interval in the terminal part of the T wave, i.e., between the peak and the end of the T wave, affect multiple ionic channels whilst drugs that are predominant IKr blockers prolong also the interval between the end of the QRS complex (i.e., the J point) and the peak of the T wave (Johannesen et al., 2014, 2016b; Vicente et al., 2016). This leads to the suggestion that drugs that are found to prolong the QT interval should have their effects on the JTp interval also determined.

### Study Design

Based on these considerations, the present study considered several different signal matrices of combination of ECG leads with the aim of determining a matrix that would lead to JTp interval measurements most stable for the purposes of serial comparisons. In practice, serial comparisons are, as already stated, bound to lead to evaluations of ECGs with different underlying heart rate. Therefore, the stability of the measurements was judged by the tightness of the JTp/heart rate relationship. To investigate signal matrices used in the determination of T wave peak, the study used long-term ECG recordings of a large population of healthy subjects. The comparison of the matrices was based on the intra-subject variability of individually heart rate corrected JTp intervals. For serial comparisons of JTp intervals, this intra-subject variability needs to be minimized for the same reasons as the intra-subject variability of QTc intervals needs to be minimized for successful serial comparisons of heart rate corrected QT intervals (Garnett et al., 2012).

Previously, it was also observed that similar distinction between predominant IKr blockers and multichannel blockers was also possible based on the interval between the J point and the center-point of T wave area (Vicente et al., 2017). Therefore, in addition to the JTp intervals, this study also investigated these intervals.

## Materials and Methods

### Population

The available data originated from two clinical pharmacology studies conducted in healthy subjects. Repeated 12-lead day-time Holter recordings were made in all study subjects while they were on no treatment and free of alcohol and/or caffeinated drinks ingestion. All subjects had a normal screening ECG and normal clinical investigation usual in clinical pharmacology studies (ICH Guideline, 2001).

### Electrocardiographic Recordings

The 12-lead Holter recordings were obtained using SEER MC (GE Healthcare, Milwaukee, WI, United States) or H12 (Mortara Instrument, Milwaukee, WI, United States) recorders, both using Mason-Likar electrode positions. Using previously described procedures (Malik et al., 2008a, 2012), multiple ECG interval measurements were made in the day-time portion of each 12-lead Holter recording. The measurements used representative morphologies derived from 10-s portions of the Holter recordings and were sampled at 1000 Hz. J point was defined as the global QRS offset, i.e., the latest QRS offset in any of the 12 leads. Likewise, QRS onset (Q point) was defined as the earliest QRS onset and T wave end as the latest T wave offset in all 12 leads. Both points were determined based on the superimposition of representative morphologies of all 12-leads superimposed on the same isoelectric axis.

In more detail (Malik et al., 2008a, 2012), the continuous 12-lead Holters were divided into 10-s segments. In each segment and each ECG lead, baseline wander was removed, and representative complex constructed by sample-by-sample medians of superimposed P-QRS-T morphological patterns of different beats. Using a combination of threshold, tangent, and pattern recognition algorithms, an automatic identification of the Q, J, and T offset points was made. In each 10-s ECG segment, these measurement points were visually checked and, where necessary, manually corrected by two independently working cardiologists. In case of their disagreement, the measurement point positions were reconciled by a senior cardiologist.

Subsequently, in each subject, published pattern matching algorithms (Hnatkova et al., 2009) were also used to ensure that similar morphologies of the QRS and T wave offset were measured similarly. As previously described (Malik et al., 2012), multiple measurements were made in each recording using ECG samples preceded by both stable and variable heart rates. The measurements were made when the subjects were supine and also during free daily activity and during postural provocations. This leads to substantial heart rate spans in each subject.

For each ECG measurement, a 5-min history of RR intervals preceding the J point and T end measurements was also obtained.

### T Wave Signal Representations

As explained, to determine the T wave peak position reasonably applicable to different ECG leads, a one-dimensional representation of T wave is needed so that it can subsequently be used for the peak detection. Individual ECG leads can also be used for the peak detection, but since each lead projects the vectorcardiographic T wave loop from a different direction, individual ECG leads are not representative of complete myocardial mass.

To investigate possibilities of one-dimensional T wave representation, the following seven matrices were investigated:

1. **XYZ:** The conceptually simplest way of reconstructing the T wave loop is by obtaining orthogonal ECG system. Hence, orthogonal XYZ representation of the ECG was obtained by previously published transformation that was optimized for recordings with Mason-Likar electrode positions (Guldenring et al., 2015). That is, at each sample of the original ECG between the J point and T end, algebraically independent leads *E*_1,8_ = {*I*,*II*,*V*_1_,*V*_2_,⋯,*V*_6_} were multiplied by a transformation matrix *T*_*8,3*_, obtaining a set of three orthogonal leads *O*_1,3_ = *E*×*T* = {*X*,*Y*,*Z*}. Using these, the orthogonal vector magnitude was obtained and used as the one-dimensional ECG representation. That is:
  $${\text{ε}}_{\text{XYZ}}={\left(X^{2}+Y^{2}+Z^{2}\right)}^{1/2}$$
2. **RMS:** Other possibility of combining different ECG leads into a unidimensional representation is by studying their distribution. All 12 leads were thus combined by calculating their root mean square, that is, at each sample of the original ECG, the one-dimensional characteristic was obtained as:
  $${\text{ε}}_{RMS}={\left(\frac{1}{12}\sum_{L\in \{I,II,III,aVR,aVL,aVF,V_{1}\cdots ,V_{6}\}}L^{2}\right)}^{1/2}$$
3. **RMS(8):** Of the 12 ECG leads, 4 leads (i.e., leads III, aVR, aVL, and aVF) are not independent and only algebraically derived from leads I and II. Therefore, the root mean square calculation of all 12 leads is potentially biased toward the signal of limb leads I and II. Therefore, the approach of root mean square was separately evaluated using only the 8 algebraically independent leads; that is:
  $${\text{ε}}_{RMS(8)}={\left(\frac{1}{8}\sum_{L\in \{I,II,V_{1}\cdots ,V_{6}\}}L^{2}\right)}^{1/2}$$
4. **Quasi-orthogonal:** Repeated discussions (Cortez et al., 2014; Ray et al., 2017) suggested that instead of orthogonal algebraic reconstruction, vectorcardiographic loops might be obtained from standard ECG leads oriented in approximately perpendicular directions. Therefore, instead of the orthogonal XYZ leads obtained from the transformation, vector magnitude was calculated using the quasi-orthogonal leads II, V_2_, and V_5_, that is:
  $${\text{ε}}_{Quasi-orthogonal}={\left(II^{2}+V_{2}^{2}+V_{5}^{2}\right)}^{1/2}$$
5. **Precordial:** To incorporate the suggestion that the distinction between the T wave upslope and downslope concerns mainly the left precordial leads, vector magnitude was calculated from leads V4, V5, and V6, i.e.,
  $${\text{ε}}_{Precordial}\text{=}{\left(V_{4}^{2}+V_{5}^{2}+V_{6}^{2}\right)}^{1/2}$$
6. **SVD(QT):** Orthogonal XYZ reconstruction by a fixed transformation assumes the same relationship between the standard 12-leads and the XYZ leads in different subjects which seems rather unlikely. Therefore, singular value decomposition was applied to the matrix of the 8 algebraically independent leads between the QRS onset and T end (Acar and Köymen, 1999). For a given ECG pattern, this provided optimized orientation of an orthogonal system of derived leads $S_{i}^{Q\,T}$, *i* = 1,2,⋯,8. Subsequently, vector magnitude was calculated from the three most dominant derived leads, i.e.,
  $${\text{ε}}_{SVD(QT)}={\left({\left(S_{1}^{QT}\right)}^{2}+{\left(S_{2}^{QT}\right)}^{2}+{\left(S_{3}^{QT}\right)}^{2}\right)}^{1/2}$$
7. **SVD(JT):** Since the singular value decomposition of the complete QT interval is substantially influenced by the power distribution within the QRS complex (Acar and Köymen, 1999), the same procedure was repeated by applying the decomposition only to the interval between the J point and the T wave offset.
  $${\text{ε}}_{SVD(JT)}={\left({\left(S_{1}^{JT}\right)}^{2}+{\left(S_{2}^{JT}\right)}^{2}+{\left(S_{3}^{JT}\right)}^{2}\right)}^{1/2}$$

### T Peak and T Median Measurements

In each ECG, each of the seven characteristics ε__▀__ (ranging from ε_***RMS***_ to ε_***SVD(JT)***_) was processed, between the J point and T end point, by the previously published algorithm to detect the peak of the T wave (Johannesen et al., 2016a). This resulted in seven different estimates of the JTp interval.

**FIGURE 1:**
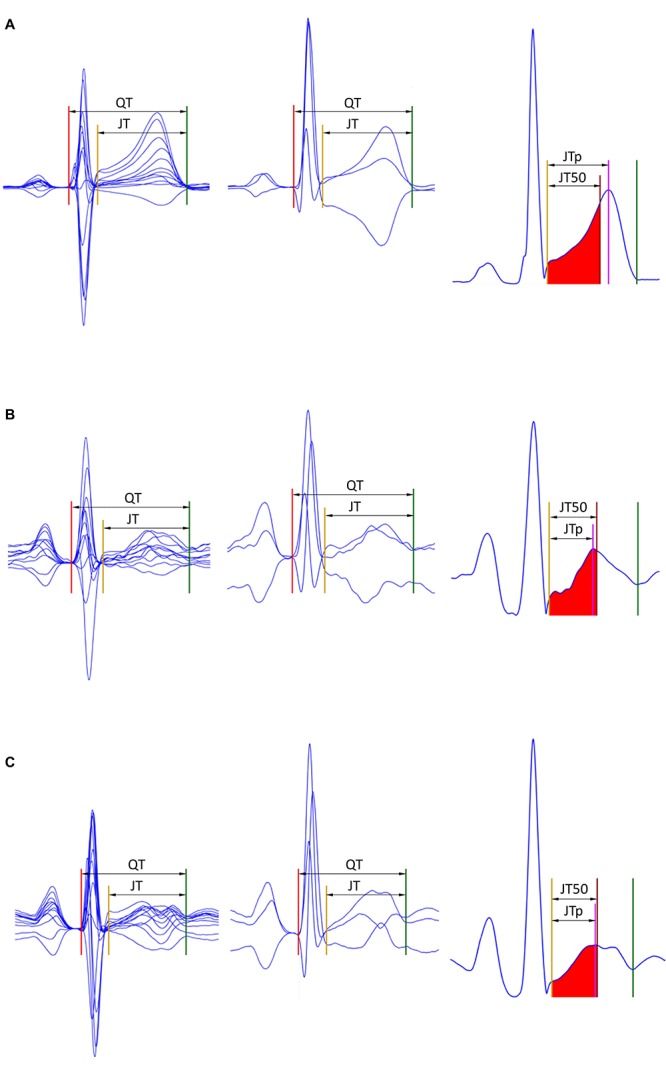
Examples of the JTp and JT50 measurements. The rows **(A–C)** of to three different ECG recordings made in a male (32 years), female (22 years), and female (42 years), respectively. The underlying heart rates were 68, 95, and 96 beats per minute, respectively. In the left panels, representative P-QRS-T complexes are shown for all 12 leads superimposed on the same isoelectric axis, the middle panels show derived orthogonal XYZ leads also on the same isoelectric axis, and the right panels show the vector magnitude of the orthogonal XYZ leads. The horizontal (time) scale of all the panels is the same. The red, yellow, and green lines show the positions of the QRS onset, QRS offset, and T offset points. In the left panels, the violet line shows the T wave peak while the red-filled area (between the yellow and brown lines) is the first middle of the area under the T wave representation (i.e., the brown line divides the area under the T wave between the yellow and green lines into two equal halves). Measurements of the JTp and JT50 intervals are shown in the left panels. Note that JTp > JT50 in panel **(A)** while JTp < JT50 in panels **(B,C)** albeit the difference between JTp and JT50 is minimal in panel **(C)**. Note also that in all three cases, the position of the peak of the T wave is different in different 12 leads as well as in different orthogonal leads.

Subsequently, the area under each of the ε__▀__ characteristic between the J point and the T end point was divided into two halves with identical areas (Hnatkova et al., 2017; Vicente et al., 2017). That is, the T wave “median” point T50 was found for which $\int_{J}^{T\,50}\varepsilon_{■}\,\left(t\right)\,dt=\int_{T\,50}^{T\,e\,n\,d}\varepsilon_{■}\,\left(t\right)\,dt$. This resulted in seven different estimates of the JT50 interval (Figure 1).

### Heart Rate Correction

For each estimate of JTp and JT50 intervals and for each study subject, all the measurements made in the given subject were related to the underlying heart rate using the previously published curvilinear regression model (Malik et al., 2013):

$${ℑ}_{i}=\alpha +\frac{\delta }{\gamma }\,\left({\bar{R\,R_{i}}}^{\gamma }-1\right)+\epsilon_{i}$$

where *ℑ*_*i*_ are the JTp or JT50 measurements in seconds, α is a central value of the measurement at underlying heart rate of 60 beats per minute, δ and γ are the slope and the curvature of the model, *ϵ*_*i*_ are zero-centered normally distributed errors, and $\bar{R\,R_{i}}$ are hysteresis corrected RR intervals (in seconds) representative of the underlying heart rate. The hysteresis correction of the underlying RR intervals was calculated using the exponential decay model (Malik et al., 2008b) that uses a subject-specific decay time constant λ which represents the time delay of achieving 95% of the adaptation after a heart rate change (Malik et al., 2016). The parameter λ was optimized for each type of interval measurement and for each subject so that the standard deviation of the residual errors *ϵ*_*i*_ was the smallest among all possibilities of λ values.

The same approach was used to obtain subject-specific relationship of the QT and JT intervals to the underlying heart rate.

### Stability and Intra-Subject Reproducibility

When the curvilinear regression model is used to correct the measured ECG intervals for the underlying heart rate, the standard deviation of the errors *ϵ*_*i*_ represent the regression residual, i.e., the standard deviation of rate-corrected intervals (Malik et al., 2008b).

Since the study used only drug-uninfluenced ECG recordings, the regression residuals are a valid estimate of intra-subject reproducibility of rate corrected intervals used in serial comparisons. Therefore, the comparison of the seven different ECG matrices (and comparison between JTp and JT50 intervals) was based on the value of the curvilinear regression residuals. In other words, to find the most stable JTp and JT50 expressions, this investigation aimed at identifying the ECG matrix that led to the lowest regression residuals among the seven possibilities.

### Statistics

Differences between JTp and JT50 intervals obtained from the different ECG matrices were displayed using density plots. The differences between the residuals of the curvilinear JTp/RR and JT50/RR regression were displayed by means of Bland-Altman plots, and their significances were evaluated by paired *t*-test. Paired *t*-test was also used to compare the residuals of JTp/RR and JT50/RR regressions with the residuals of QT/RR and JT/RR regressions, as well as to compare the corresponding hysteresis time constants and the slope and curvature parameters of the curvilinear regressions.

Data are presented as mean ± standard deviation. In addition to the total study population, separate evaluation was made for female and male subjects. Nevertheless, the principal outcome of the study, i.e., the identification of the optimal ECG matrix, was made based on the complete study population. A *p*-value below 0.05 was considered statistically significant without adjustment for multiplicity.

## Results

### Population

Holter ECG recordings were obtained in 523 healthy subjects aged 33.5 ± 8.4 years (range 18.1–55.4 years). Of these, 254 (48.6%) were females. A total of 236 (45.1%) and 259 (49.5%) study subjects identified themselves as of Black/African origin or White/Caucasians, respectively. The remainder classified themselves as Asian, Polar region natives, or “Other” race.

### ECG Data

In the Holter recordings, altogether 660,657 ECG measurements of the J point to T end intervals were made. Of these, technical suitability check based on objective noise assessment (Batchvarov et al., 2002) excluded 3,523 ECGs (0.53%) that were too noise-polluted for reasonable T peak assessment. The remaining 657,134 measurements were accepted for the data analyses. On average, there were 1,256 ± 220 accepted ECG measurements per subject (inter-quartile range 1,058–1,436).

### Measurement Comparisons

**FIGURE 2:**
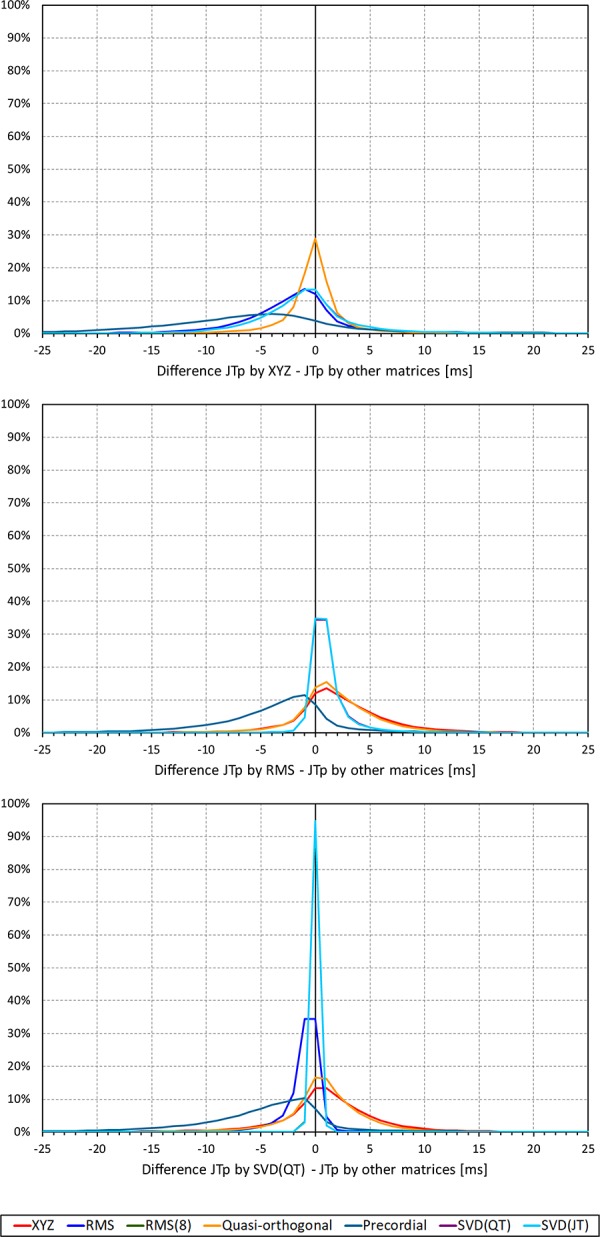
Sample density distributions of the differences between JTp intervals measured based on different one-dimensional matrices in all ECG samples. The top, middle, and bottom panels show the differences between JTp intervals measured based on all other matrices and the measurements based on the XYZ, RMS, and SVD(QT) matrix, respectively. The graphs were constructed using 1 ms bins. Note that in some cases, the lines were superimposed and that some of the graphs might be hidden bellow others – e.g., within the precision of the graphics, the results of RMS(8) were practically identical to those of SVD(JT) which is shown on the top.

**FIGURE 3:**
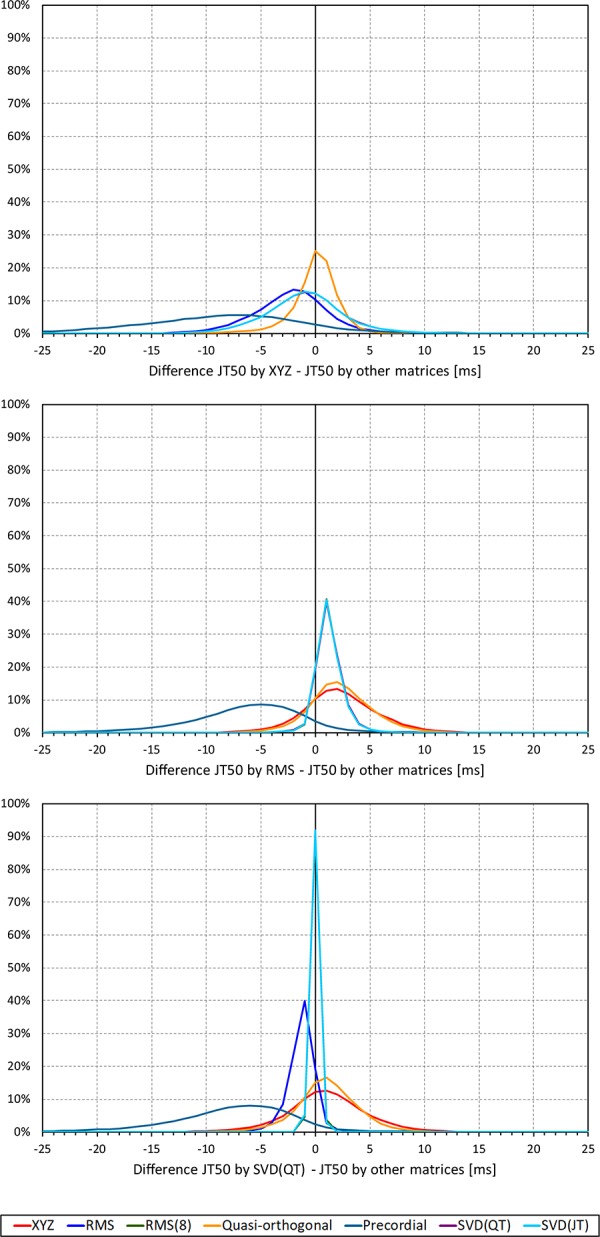
Sample density distributions of the differences between JT50 intervals measured based on different one-dimensional matrices in all ECG samples. The top, middle, and bottom panels show the differences between JT50 intervals measured based on all other matrices and the measurement based on the XYZ, RMS, and SVD(QT) matrix, respectively. The graphs were constructed using 1 ms bins. Note that in some cases, the lines were superimposed and that some of the graphs might be hidden bellow others – e.g., within the precision of the graphics, the results of RMS(8) were practically identical to those of SVD(JT) which is shown on the top.

Not surprisingly, while some large discrepancies of the JTp and JT50 measurements existed among different ECG matrices in noise-polluted ECGs, the overall numerical differences among different matrices were modest, mostly less than 10 milliseconds (Figures 2, 3). The largest differences were found between the measurements based on the precordial matrix and the other possibilities.

**FIGURE 4:**
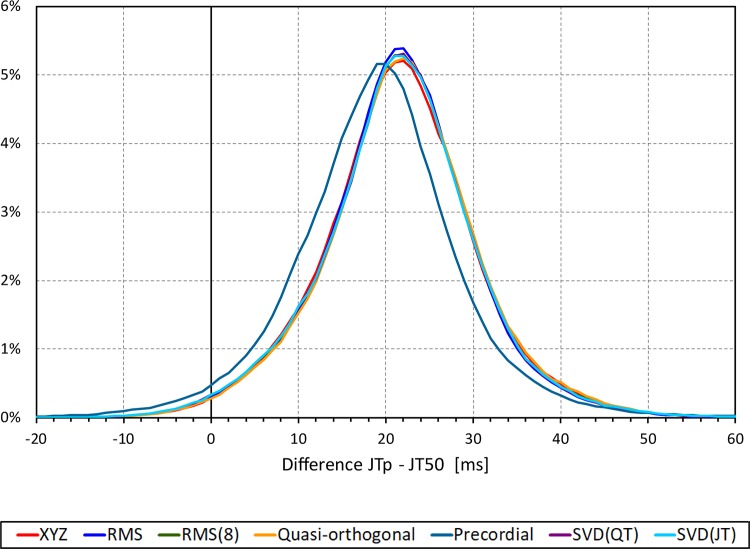
Sample density distributions of the differences between JTp and JT50 intervals measured based on different one-dimensional matrices in all ECG samples. The graphs were constructed using 1 ms bins. Note that in some cases, the lines were superimposed and that some of the graphs might be hidden bellow others – e.g., within the precision of the graphics, the results of RMS(8) were practically identical to those of SVD(JT) which is shown on the top.

**FIGURE 5:**
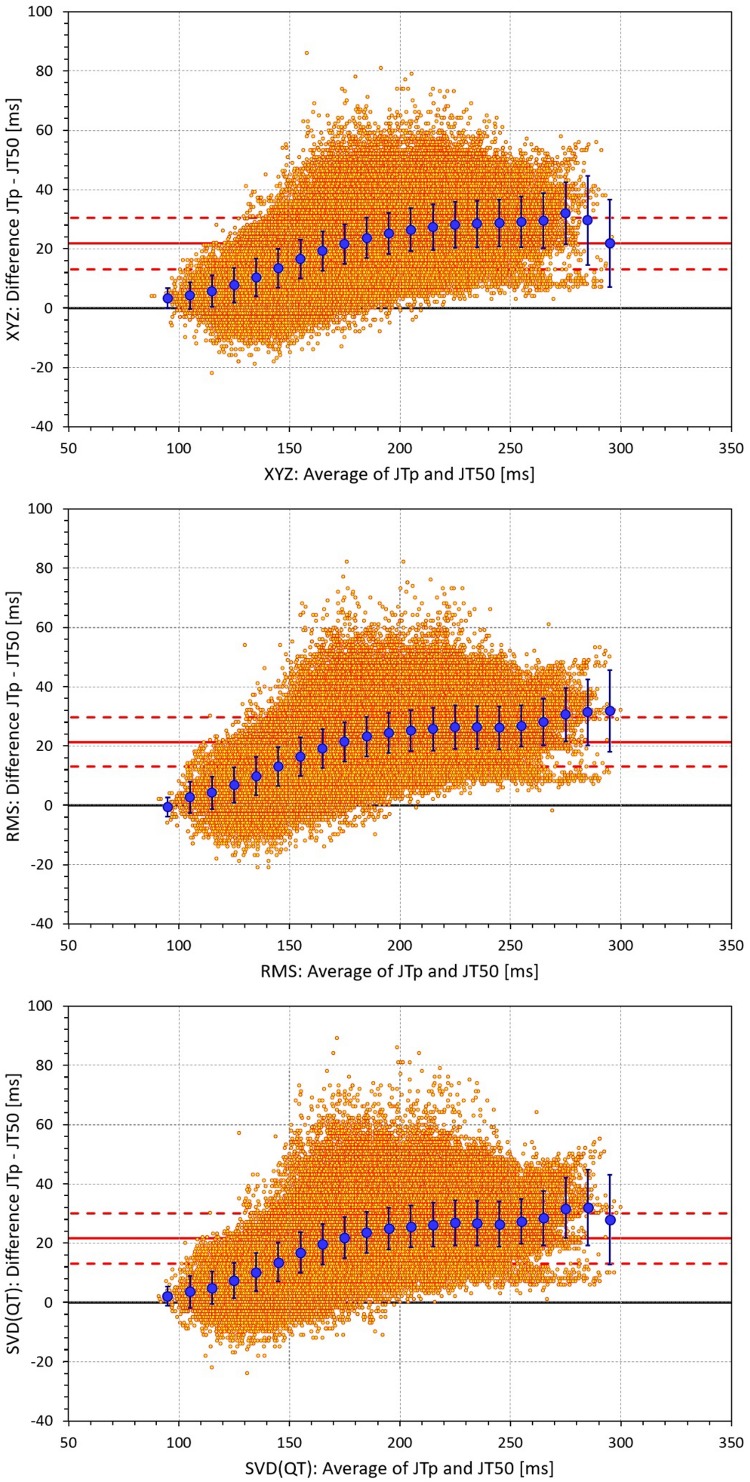
Bland-Altman plots of the individual differences between JTp and JT50 intervals based on XYZ matrix (top panel), RMS matrix (middle panel), and SVD(QT) matrix (bottom panel). In each panel, the bold red line shows the mean difference, the dashed red lines show the mean ± standard deviation. In each panel, the large blue marks show the means of the JTp – JT50 differences in 10-millisecond bins of the JTp and JT50 averages (from 90–100 ms to 290–300 ms); the blue error bars are the standard deviations of the JTp – JT50 differences in these bins.

The difference between the JTp and JT50 measurements was more substantial. As shown in Figure 4, the JTp–JT50 showed a mode slightly above 20 ms for all methods with the exception of the precordial matrix for which the mode was slightly below 20 ms. More importantly, Figure 5 shows that the JTp–JT50 difference was not without bias. At fast heart rates (when both JTp and JT50 were short), T peak occasionally preceded the T median while at slower heart rates, the relationship was systematically reversed.

### Regression Residuals of the Curvilinear Models

**FIGURE 6:**
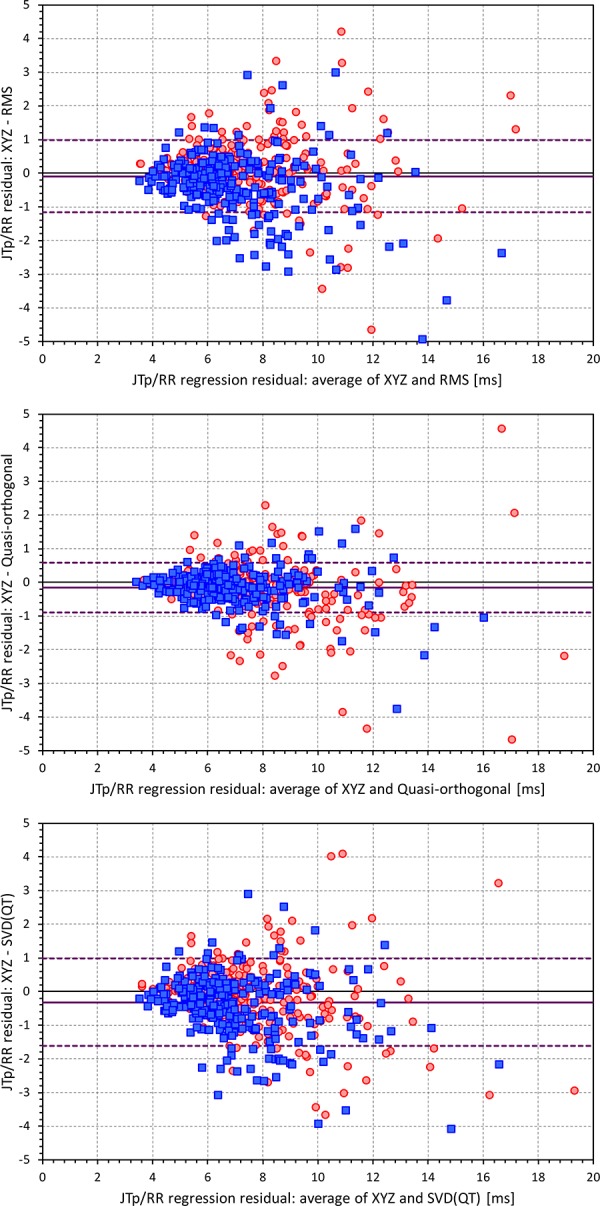
Bland-Altman plots of the differences between curvilinear JTp/RR regression residuals in individual study subjects. The top, middle, and bottom panels show the differences for JTp interval based on XYZ and RMS matrices, XYZ and Quasi-orthogonal matrices, and XYZ and SVD(QT) matrices, respectively. In each panel, the red circles and blue squares show female and male subjects, the bold violet line shows the mean difference and the dashed violet lines show the mean ± standard deviation of the differences.

**FIGURE 7:**
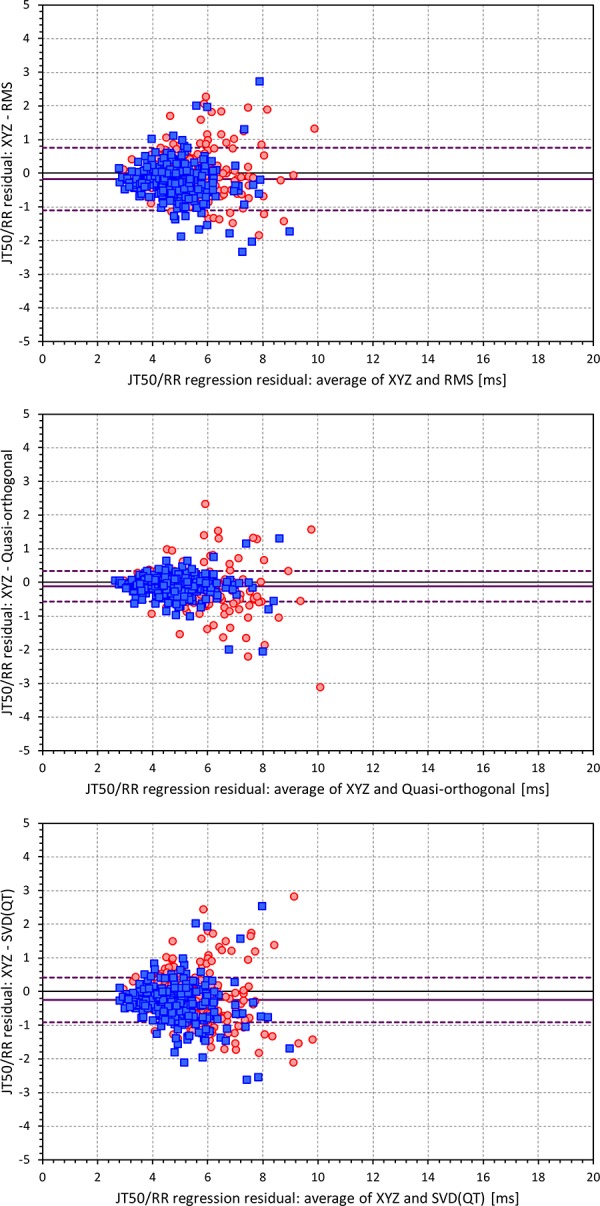
Bland-Altman plots of the differences between curvilinear JT50/RR regression residuals in individual study subjects. The top, middle, and bottom panels show the differences for JT50 interval based on XYZ and RMS matrices, XYZ and Quasi-orthogonal matrices, and XYZ and SVD(QT) matrices, respectively. In each panel, the red circles and blue squares show female and male subjects, the bold violet line shows the mean difference and the dashed violet lines show the mean ± standard deviation of the differences. Compare with Figure 6 and note the difference in the spread of the data.

**FIGURE 8:**
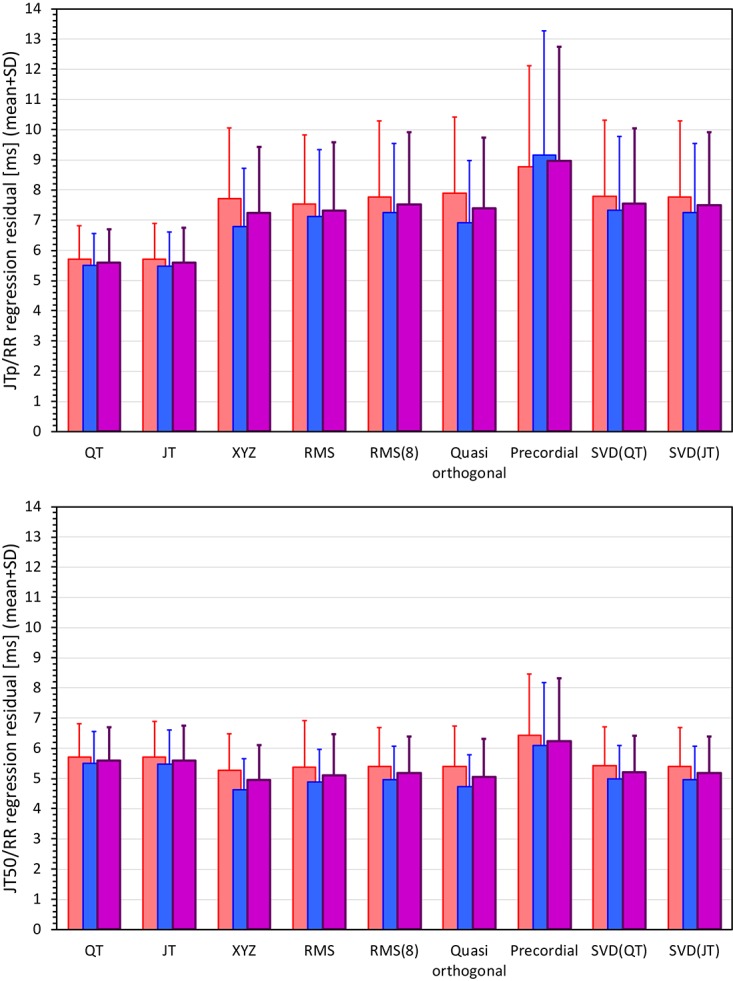
Statistical summaries of the curvilinear JTp/RR (top panel) and JT50/RR (bottom panel) regression residuals for the interval measurements based on different one-dimensional ECG matrices. For comparison, the residuals of the QT/RR and JT/RR regressions are also shown in both panels. The red, blue, and violet bars show the mean values in female, males, and the complete study population, respectively. The errors bars show the corresponding standard deviations.

Figures 6, 7 show Bland-Altman plots comparing the curvilinear regression of JTp and JT50 measurements based on the XYZ matrix with the interval measurements based on selected other ECG matrices. In all the cases shown in these figures, the measurements based on XYZ ECG representation fitted the curvilinear regression models to heart rate more tightly compared to the other matrices. As shown in Table 1 and summarized in Figure 8, XYZ representation led to higher stability of heart rate dependency of both JTp and JT50 measurements in comparison to all other investigated possibilities.

**TABLE 1:** Curvilinear regression residuals.

**Matrix**	**JTp**		**JT50**	
		
	**Residual (ms)**	***p*-value vs. XYZ**	**Residual (ms)**	***p*-value vs. XYZ**
XYZ	7.237 ± 2.190		4.940 ± 1.167	
RMS	7.325 ± 2.262	0.0299	5.116 ± 1.357	<0.0001
RMS(8)	7.510 ± 2.402	<0.0001	5.174 ± 1.221	<0.0001
Quasi-orthogonal	7.394 ± 2.344	<0.0001	5.056 ± 1.247	<0.0001
Precordial	8.963 ± 3.775	<0.0001	6.249 ± 2.067	<0.0001
SVD(QT)	7.555 ± 2.491	<0.0001	5.196 ± 1.232	<0.0001
SVD(JT)	7.510 ± 2.401	<0.0001	5.179 ± 1.223	<0.0001

*For different ECG matrices, the table shows mean ± standard deviation of the within-subject curvilinear JTp/RR and JT50/RR regression residuals. *P*-values are shown for the paired comparisons with the residuals of the XYZ matrix. Note that for all matrices, the paired comparisons between the JTp/RR and JT50/RR residuals were highly statistically significant (*p* < 0.0001 for all).*

**FIGURE 9:**
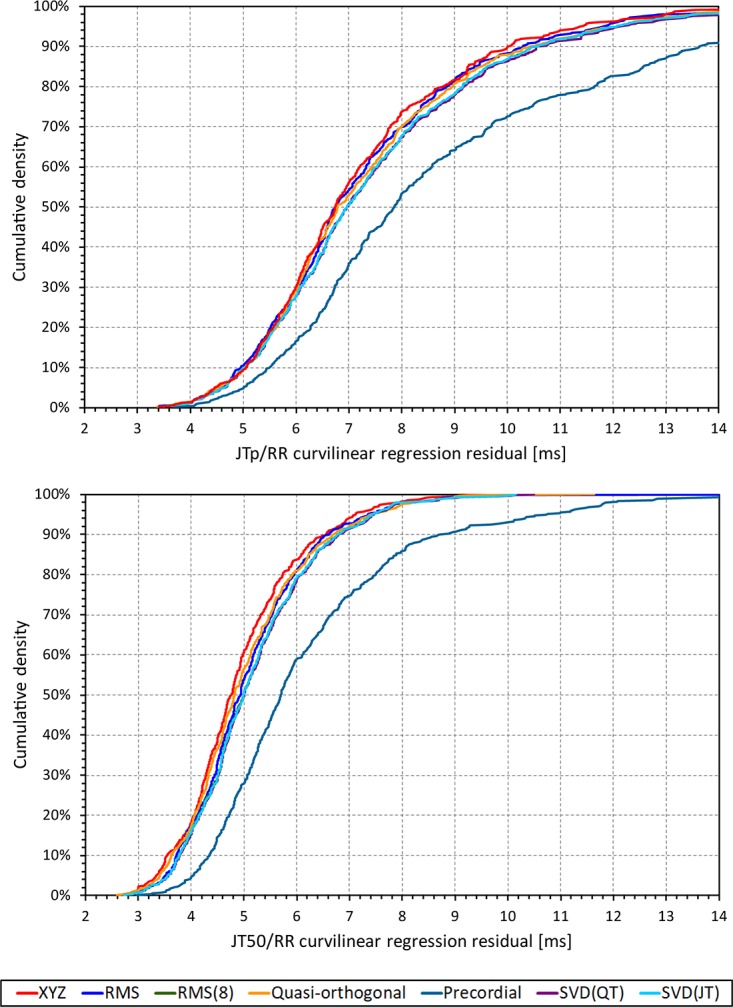
Cumulative sample distributions of the within-subject curvilinear residuals the JTp/RR (top panel) and JT50/RR (bottom panel) regressions superimposed for different ECG matrices. Note that while the red line of the XYZ matrix is on the left side of the other distributions, the differences are not particularly large (apart from the precordial matrix). Note that in some cases, the lines were superimposed and that some of the graphs might be hidden bellow others – e.g., within the precision of the graphics, the results of RMS(8) were practically identical to those of SVD(JT) which is shown on the top.

Although statistically significant, the differences among the JTp regression residuals (and similarly between the JT50 regression residuals) of different ECG matrices were small. This is also demonstrated in Figure 9 that shows the cumulative distributions of the regression residuals in study subjects.

More importantly, however, the regression residuals of JT50 intervals were non-trivially smaller compared to those of JTp intervals. For all ECG matrices, the differences among the JT50 and JTp residuals shown in Table 1 were highly statistically significant (*p* < 0.0001 in all cases). This suggests that the measurement of the median point of T wave area was systematically made in a more stable and reproducible way than that of the peak of the T wave.

**FIGURE 10:**
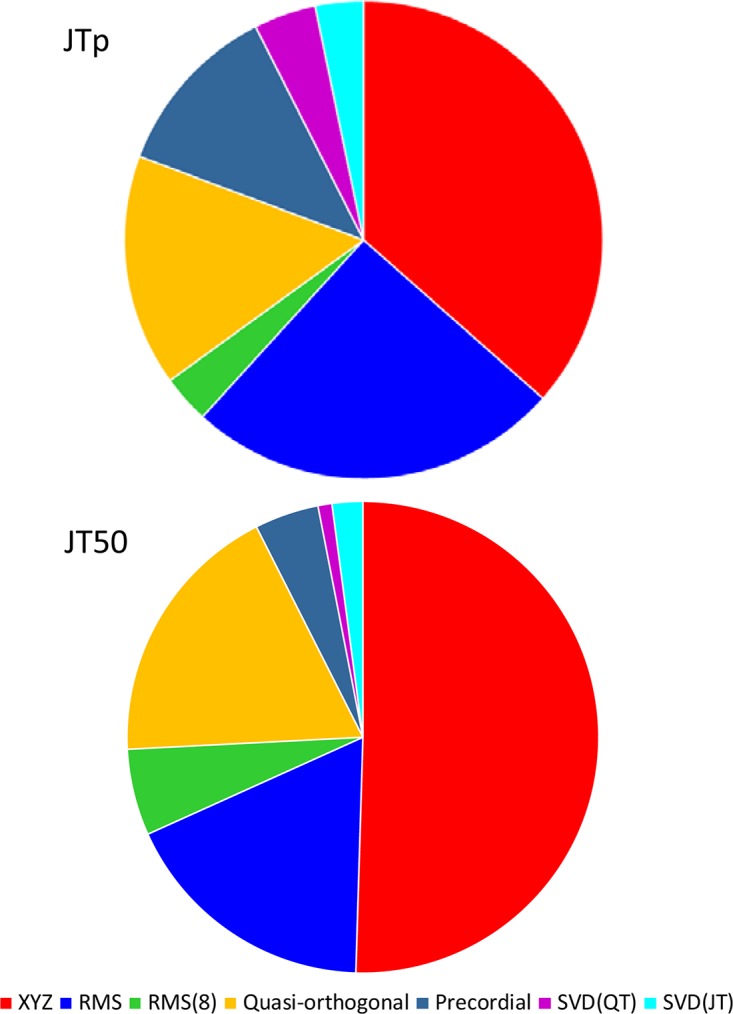
Distribution of the one-dimensional ECG matrices that led to the intra-subject lowest residuals for the JTp/RR (top panel) and JT50/RR (bottom panel) regressions. The pie charts show the proportions of the subjects in whom the given one-dimensional matrix led to the lowest regression residual.

While the interval measurements based on the XYZ matrix were, in terms of the regression to the underlying heart rate, the most stable in the overall population, this was not necessarily the case in all study subjects considered separately. Figure 10 shows the distribution of ECG matrices that showed the lowest curvilinear regression residuals in individual subjects. The XYZ ECG matrix led to the lowest residuals of the JTp and JT50 intervals in 191 (36.5%) and 264 (50.5%) subjects, respectively. For the RMS matrix, the corresponding numbers were 132 (25.2%) and 93 (17.8%) subjects while for the Quasi-orthogonal matrix, the numbers were 82 (15.7%) and 96 (18.4%) subjects.

### Comparison With QT and JT Intervals

The residuals of the curvilinear regressions between the QT and JT intervals and RR intervals of the underlying heart rate were 5.6 ± 1.1 ms and 5.6 ± 1.2 ms, respectively (no statistical difference between the residuals of the QT and JT intervals).

The residuals of JTp intervals based on the XYZ ECG matrix (see Table 1) were statistically significantly larger than those of QT and JT intervals. On the contrary, the residuals of JT50 intervals were statistically significantly smaller than those of QT and JT intervals (*p* < 0.0001 for all these comparisons).

The curvilinear slopes (parameters δ of the regression) of the QT, JT, JTp, and JT50 intervals (XYZ matrix) were 0.150 ± 0.029, 0.149 ± 0.030, 0.145 ± 0.070, and 0.121 ± 0.046, respectively. There were no statistical differences among the QT, JT and JTp slopes, but the curvilinear regressions of the JT50 intervals were statistically significantly less steep that those of the other intervals (*p* < 0.0001 for all comparisons).

Differences also existed in the curvatures (parameters γ) of the curvilinear regressions. For the QT, JT, JTp, and JT50 intervals (XYZ matrix) these were 0.72 ± 0.69, 0.71 ± 0.66, 0.52 ± 1.04, and 1.00 ± 1.10, respectively. While the JTp/RR regressions were more curved than all others, the JT50 regressions were less curved than all others and, on average, very close to simple linear regressions (*p* < 0.0001 for all comparisons).

Finally, differences existed in the hysteresis time constants λ, i.e., the delays needed for the intervals to achieve 95% adaptation after a heart rate change. For the QT, JT, JTp and JT50 intervals (XYZ matrix) these were 116.0 ± 21.5, 121.5 ± 23.2, 134.3 ± 32.0, and 105.2 ± 25.0 s, respectively. All their comparisons were statistically significant (*p* < 0.0001 for all), meaning that the JT and JTp intervals adapted more slowly than the QT interval while the adaptation of the JT50 interval was faster compared to the QT interval.

## Discussion

The study leads to several conclusions of methodological importance. There were only numerically tiny differences among the JTp and among the JT50 intervals measured using different one-dimensional ECG matrices. The XYZ matrix (Guldenring et al., 2015) that was found to have the lowest regression residuals is easily applicable to all 12-lead ECGs recorded with Mason-Likar electrode positions. Since Mason-Likar configuration is the standard for long-term ECG recordings that are presently used in the majority of pharmaceutical studies, the XYZ matrix may be proposed for future studies of JTp and JT50 intervals. Incidentally, the XYZ matrix was also used in the seminal study that confirmed the importance of JTp interval investigations for the classification of QT prolonging drugs (Johannesen et al., 2016a; Vicente et al., 2016).

This observation of only small differences and of the XYZ matrix preference is potentially surprising. When designing the study, we expected to find the residuals of the individually optimized matrices, i.e., the SVD(QT) or SVD(JT) expression, to suppress the variability of the “universal” expressions substantially. This was not the case probably because singular value decomposition optimizes the rectangular projection for individual ECG samples (Acar and Köymen, 1999) which means that the ECG lead transformations are unlikely constant for all ECGs of the same subject. The within-subject variability of the transformations will lead to unfavorable results compared to a “universal” transformation such as that of the XYZ matrix.

The present study also observed that compared to JTp intervals, the JT50 intervals lead to substantially lower residuals of their intra-subject relationship to heart rate. This is in agreement with the previous observations that also found reduction in data variability when using JT50 intervals (Vicente et al., 2017). Hence, if the importance of JT50 interval for the classification of QT prolonging drugs (Vicente et al., 2017) is confirmed in future studies, the use of this interval would become preferable instead of the JTp interval because of the lower measurement variability. As already explained, the residuals of the intra-subject regression to the underlying RR intervals equal to the standard deviation of individually heart rate corrected intervals. If similar reduction in the variability as we have observed is also found for the variability of placebo-to-baseline differences, power sample calculations would clearly prefer the use of JT50 rather than JTp intervals (Malik et al., 2004; Zhang and Machado, 2008).

In some cases (e.g., all ECGs shown in Figure 1), the value of the one-dimensional ECG representation at the J and T offset points is greater than 0. We have therefore also investigated the possibility of measuring the point of the middle of the area between the T wave and the line connecting the ECG representation value at the J and T offset points (leading to JT50’ measurements). While the JT50’/RR residuals (results not shown) were still significantly lower than the JTp/RR residuals, they were also significantly larger than the JT50/RR residuals that we present.

In addition to drug-classification studies, JTp interval was also shown to carry prognostic mortality information (O’Neal et al., 2017). It would be interesting to investigate whether similar prognostic information can be obtained using the JT50 interval.

The curvilinear regression residuals, i.e., the heart-rate independent variability, of the JTp intervals were larger than those of the QT intervals. This means that measurement of the T peak is less stable than the measurement of the end of the T wave. While this might be somewhat counterintuitive, it corresponds to the experience of T wave pattern variability due to respiration, meal intake, and other processes that influence the position of the heart within the thorax. On the contrary, the residuals of the JT50 intervals were lower than those of the QT interval. This is not surprising since the median point of the area under the one-dimensional representation is little influenced by imprecision at the J point or at the end of the T wave since the voltages are generally rather low at these points.

The increased curvature of the JTp/RR patterns compared to the QT/RR and JT50/RR patterns likely relates to the shifts toward more symmetrical T waves at faster heart rates. The JT50/RR patterns were, on average, practically linear, i.e., less curved compared to the QT/RR patterns. While this, together with the shallower curvilinear slopes, might also be related to the shifts in the T wave patterns, it needs to be noted that the standard deviation of the curvatures over the investigated population was large and that the population mean values might potentially be misleading in individual cases.

Finally, while the study found statistically significant differences among the RR-interval hysteresis time constants, the numerical differences were small and only little different from 2 min. Hence, unless dealing with situations of substantial and very abrupt heart rate changes [e.g., those seen after administration of some contrast agents (Malik et al., 2009)] the previously proposed universal model of RR-interval hysteresis with the 2-min time constant (Malik et al., 2016) will likely work for JTp/RR and JT50/RR hysteresis equally well as for the QT/RR hysteresis.

### Limitations

Limitations of the study also need to be acknowledged. Many other one-dimensional ECG matrices are possible. Nevertheless, since we have found little differences among those tested and since the most stable results were found with “universal” matrix construction, it seems unlikely that some specific matrix would have performed substantially better compared to the XYZ representation. The XYZ matrix was designed for ECGs recorded with Mason-Likar electrode positions. It seems reasonable to propose that for ECGs recorded using other standard electrode positions, corresponding XYZ transformations (Kors et al., 1990) would also show optimal results, but this has not been tested. The study analyzed drug-free data. It may be that different one-dimensional ECG matrices would perform differently in categorizing the effects of different drugs. We are unable to comment on such a possibility. Likewise, we are unable to answer the question of whether these results obtained in healthy and relatively young subjects with normal physiologic ECGs would also be applicable to the elderly or to patients with congenital or other repolarisation abnormalities. Previous studies also related repolarisation ECG intervals to intra-myocardial repolarisation heterogeneity (Brisinda et al., 2004; Meijborg et al., 2017). Since we did not have any independent measurements of intra-myocardial electrophysiology processes available in the healthy subjects of this study, we were unable to study any such relationship. Finally, while the selection of the T wave peak measurement by minimizing the rate-corrected JTp variability is appropriate to the purposes of serial JTp comparisons, it might not be optimal for other applications of the interval measurements such as the beat-to-beat variability if these were eventually found of interest.

## Conclusion and Practical Implications

Searching for optimum ECG representation to be used when categorizing QT prolonging drugs appears unnecessary. The standard ECG conversion into orthogonal leads and calculating vector magnitude or the orthogonal leads appears universally applicable.

Testing of the JT50 interval changes should be carried out in clinical studies that used the JTp interval analyses. If the drug classification possibility of the JT50 interval is confirmed, its lower variability would make it a good replacement of the JTp interval.

## Data Availability

All datasets generated for this study are included in the manuscript.

## Ethics Statement

Both the original studies were approved by the relevant ethics boards and all participants gave written informed consent in accordance with the Helsinki declaration. Since only anonymized off-treatment data are presented here, the details of the source studies are of no relevance. For the same reason, no separate ethics clearance of the present investigation was required as per the local legislation.

## Author Contributions

All authors listed have made a substantial, direct and intellectual contribution to the work, and approved it for publication.

## Conflict of Interest Statement

The authors declare that the research was conducted in the absence of any commercial or financial relationships that could be construed as a potential conflict of interest.
